# Supplementary material for: Association between Dietary Diversity Changes and Cognitive Impairment among Older People: Findings from a Nationwide Cohort Study
Source: Nutrients. 2022 Mar 16;14(6):1251. doi: 10.3390/nu14061251 (PMC8952508; doi:10.3390/nu14061251)
Supplement: Supplementary file 1 [file nutrients-14-01251-s001.zip › nutrients-1574340-supplementary.pdf]

Supplement Materials

Supplemental Figure S1. Cohort Selection Criteria, CLHLS Survey form 1998-2014 2

Supplemental Table S1. The numbers (percentages) of participants with missing covariates 3

Supplemental Table S2. Baseline characteristics of older people according to absolute DDS change patterns (n=9,726) 4

Supplemental Table S3. The association between DDS change patterns and cognitive impairment 6

Supplemental Table S4. The association between absolute DDS change groups and cognitive impairment in plant-based and animal-based DDS 8

Supplemental Table S5. The incidence rate of cognitive impairment among DDS change patterns in subgroups 9

Supplemental Table S6. Sensitivity analyses of the association between DDS change patterns and cognitive impairment 11

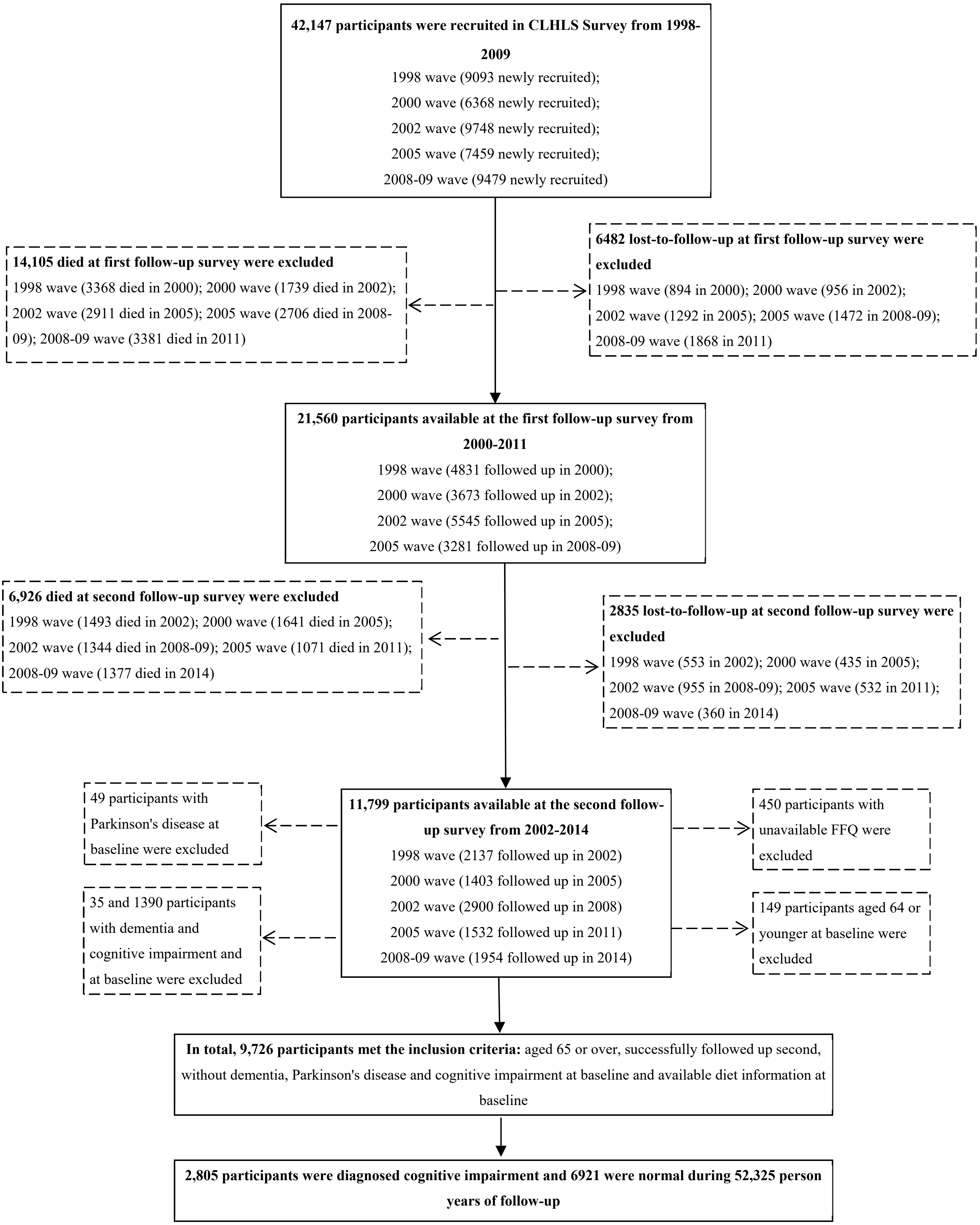

Supplemental Figure S1. Cohort Selection Criteria, CLHLS Survey form 1998-2014

Abbreviations: Chinese Longitudinal Healthy Longevity Study (CLHL)

Supplemental Table S1. The numbers (percentages) of participants with missing covariates

**Covariates**	**n**	**%**
Occupation	7	0.1
Live pattern	1	0.0
Tobacco smoking	7	0.1
Alcohol drinking	11	0.1
Use of artificial denture	7	0.1
BMI	154	1.6
Hypertension	68	0.7
Diabetes	77	0.8
Heart diseases	69	0.7
Cerebrovascular diseases	73	0.8
Respiratory diseases	74	0.8
Digestive system diseases	76	0.8
Cancer	82	0.8
Eye diseases	65	0.7
Arthritis	1	0.0

Supplemental Table S2. Baseline characteristics of older people according to absolute DDS change patterns (n=9,726)

Characteristics	Absolute DDS changes from baseline to first follow up				
	Extreme decline	Moderate decline	Stable	Moderate improvement	Large improvement
**Participants**	1026 (10.6)	2027 (20.8)	3250 (33.4)	1777 (18.3)	1646 (16.9)
**Age in years, mean (SD)**	79.6 (10.1)	80.0 (10.1)	80.4 (10.2)	80.1 (10.0)	79.5 (10.1)
**Male**	472 (46.0)	979 (48.3)	1555 (47.9)	857 (48.2)	781 (47.5)
**Urban Residence**	230 (22.4)	457 (22.6)	827 (25.5)	424 (23.9)	400 (24.3)
**Education level**					
No schooling	550 (53.6)	1123 (55.4)	1754 (54.0)	982 (55.3)	919 (55.8)
≤6 years	372 (36.3)	682 (33.7)	1126 (34.7)	594 (33.4)	542 (32.9)
>6 years	104 (10.1)	222 (11.0)	370 (11.4)	201 (11.3)	185 (11.2)
**Occupation**					
Worker	252 (24.6)	526 (26.0)	950 (29.3)	542 (30.5)	437 (26.6)
Farmer	521 (50.8)	972 (48.0)	1411 (43.5)	801 (45.1)	778 (47.3)
Others	253 (24.7)	528 (26.1)	886 (27.3)	432 (24.3)	430 (26.1)
**Source of income**					
Pension	210 (20.5)	405 (20.0)	740 (22.8)	396 (22.3)	333 (20.2)
Other	816 (79.5)	1622 (80.0)	2510 (77.2)	1381 (77.7)	1313 (79.8)
**In marriage**	455 (44.4)	892 (44.0)	1427 (43.9)	808 (45.5)	748 (45.4)
**Living pattern**					
Living with family members	865 (84.3)	1720 (84.9)	2712 (83.5)	1496 (84.2)	1373 (83.5)
Alone	139 (13.6)	267 (13.2)	454 (14.0)	238 (13.4)	227 (13.8)
At nursing home	22 (2.1)	40 (2.0)	84 (2.6)	43 (2.4)	45 (2.7)
**Tobacco smoking**					
Current smoker	241 (23.5)	492 (24.3)	732 (22.5)	409 (23.0)	421 (25.6)
Former smoker	111 (10.8)	276 (13.6)	450 (13.9)	243 (13.7)	230 (14.0)
Non-smoker	672 (65.6)	1258 (62.1)	2065 (63.6)	1125 (63.3)	994 (60.4)
**Alcohol drinking**					
Current drinker	250 (24.4)	531 (26.2)	790 (24.4)	442 (24.9)	378 (23.0)
Former drinker	83 (8.1)	197 (9.7)	254 (7.8)	174 (9.8)	162 (9.9)
Non-drinker	693 (67.5)	1298 (64.1)	2197 (67.8)	1161 (65.3)	1105 (67.2)
**Regular exercises**	402 (39.2)	763 (37.6)	1244 (38.3)	629 (35.4)	548 (33.3)
**Use of artificial denture**	321 (31.3)	582 (28.7)	993 (30.6)	549 (30.9)	506 (30.8)
**BMI, mean (SD), kg/m^2**	22.1 (4.7)	21.9 (4.4)	22.0 (4.9)	21.7 (4.4)	21.4 (4.2)

**Chronic diseases**					
Hypertension	168 (16.6)	327 (16.3)	545 (16.9)	293 (16.6)	310 (18.9)
Diabetes	20 (2.0)	45 (2.2)	56 (1.7)	35 (2.0)	37 (2.3)
Heart diseases	83 (8.2)	149 (7.4)	252 (7.8)	144 (8.2)	146 (8.9)
Cerebrovascular diseases	37 (3.7)	71 (3.5)	97 (3.0)	66 (3.8)	72 (4.4)
Respiratory diseases	104 (10.3)	203 (10.1)	337 (10.5)	163 (9.2)	184 (11.2)
Digestive diseases	51 (5.0)	111 (5.5)	155 (4.8)	96 (5.5)	80 (4.9)
Cancer	2 (0.2)	5 (0.3)	11 (0.3)	2 (0.1)	3 (0.2)
ADL disabled	67 (6.5)	169 (8.3)	258 (7.9)	143 (8.1)	125 (7.6)
Eye diseases	95 (9.4)	216 (10.7)	372 (11.5)	179 (10.1)	173 (10.6)
Arthritis	192 (18.7)	364 (18.0)	551 (17.0)	324 (18.2)	277 (16.8)

Values are n (%) or mean (standard deviation, SD). DDS: dietary diversity score; BMI: body mass index; ADL: activities of daily living.

Supplemental Table S3. The association between DDS change patterns and cognitive impairment

DDS patterns	DDS change patterns from baseline to first follow up								
	High-High	High-Medium	High-Low	Medium-High	Medium-Medium	Medium-Low	Low-High	Low-Medium	Low-Low
**Overall**									
No of death/ person years	239/6941	321/6557	37/649	359/8030	1219/21165	259/3815	38/775	218/3112	115/1282
Mortality rate	34.4	49.0	57.0	44.7	57.6	67.9	49.0	70.0	89.7
Model 1	1.00 (ref)	1.29 (1.09-1.52)	1.22 (0.86-1.73)	1.07 (0.91-1.26)	1.56 (1.35-1.79)	1.88 (1.57-2.24)	1.01 (0.72-1.43)	2.16 (1.79-2.60)	2.52 (2.02-3.16)
Model 2	1.00 (ref)	1.34 (1.13-1.59)	1.35 (0.95-1.91)	1.13 (0.96-1.33)	1.69 (1.46-1.94)	2.13 (1.77-2.55)	1.19 (0.84-1.68)	2.40 (1.99-2.90)	2.92 (2.32-3.67)
Model 3	1.00 (ref)	1.33 (1.12-1.57)	1.38 (0.98-1.96)	1.11 (0.94-1.32)	1.61 (1.39-1.86)	2.00 (1.66-2.40)	1.10 (0.78-1.56)	2.30 (1.90-2.78)	2.80 (2.23-3.53)
**Plant-based DDS**									
No of death/ person years	191/5167	308/6608	58/873	295/7340	1101/20065	322/5041	45/823	285/4194	200/2213
Mortality rate	37.0	46.6	66.4	40.2	54.9	63.9	54.7	68.0	90.4
Model 1	1 (ref)	1.20 (1.00-1.44)	1.45 (1.08-1.94)	0.94 (0.78-1.12)	1.35 (1.16-1.58)	1.49 (1.24-1.79)	1.11 (0.80-1.54)	1.69 (1.40-2.03)	2.14 (1.75-2.62)
Model 2	1 (ref)	1.24 (1.04-1.49)	1.43 (1.07-1.93)	0.99 (0.82-1.18)	1.46 (1.25-1.70)	1.67 (1.39-2.00)	1.26 (0.91-1.74)	1.85 (1.54-2.23)	2.34 (1.91-2.87)
Model 3	1 (ref)	1.21 (1.01-1.45)	1.49 (1.11-2.01)	0.91 (0.76-1.10)	1.31 (1.12-1.54)	1.44 (1.19-1.73)	1.02 (0.73-1.42)	1.55 (1.28-1.88)	2.00 (1.62-2.46)
**Animal-based DDS**									

No of death/ person years	326/7806	251/5626	80/1557	363/7469	714/12267	363/5507	99/2277	327/5434	282/4381
Mortality rate	41.8	44.6	51.4	48.6	58.2	65.9	43.5	60.2	64.4
Model 1	1 (ref)	1.11 (0.94-1.31)	1.25 (0.98-1.60)	1.06 (0.92-1.23)	1.58 (1.39-1.80)	1.70 (1.46-1.97)	0.99 (0.79-1.24)	1.63 (1.40-1.90)	1.88 (1.60-2.21)
Model 2	1 (ref)	1.18 (1.00-1.40)	1.32 (1.04-1.69)	1.15 (0.99-1.34)	1.71 (1.50-1.96)	1.91 (1.64-2.23)	1.06 (0.85-1.33)	1.82 (1.55-2.13)	2.12 (1.80-2.49)
Model 3	1 (ref)	1.20 (1.02-1.42)	1.30 (1.01-1.66)	1.07 (0.92-1.25)	1.57 (1.37-1.80)	1.69 (1.44-1.97)	0.99 (0.79-1.24)	1.61 (1.37-1.90)	1.89 (1.60-2.24)

DDS: dietary diversity score; ADL: activities of daily living; Incidence rate (1,000 person years).

Model 1: Adjusted for age (continuous) and sex.

Model 2: Adjusted for model 1 plus residence, educational level, occupation and source of income.

Model 3: Adjusted for model 2 plus current marital status, living pattern, tobacco smoking, alcohol drinking, regular exercise, BMI (continuous), use of artificial denture, hypertension, diabetes, heart disease, cerebrovascular disease, respiratory disease, digestive system diseases, cancer, eye disease, arthritis and ADL disabled.

Plant-based DDS and animal-based DDS were mutually adjusted.

Supplemental Table S4. The association between absolute DDS change groups and cognitive impairment in plant-based and animal-based DDS

**Absolute DDS change patterns**	Extreme decline	Moderate decline	Stable	Moderate improvement	Large improvement
Plant-based DDS	0.87 (0.75-1.01)	0.93 (0.83-1.04)	1.00 (ref)	0.92 (0.82-1.03)	0.82 (0.70-0.96)
Animal-based DDS	1.00 (0.86-1.16)	0.98 (0.88-1.08)	1.00 (ref)	0.91 (0.82-1.00)	0.83 (0.72-0.96)

DDS: dietary diversity score; BMI: body mass index.

Adjusted for age (continuous), sex, residence, educational level, occupation, source of income, current marital status, living pattern, tobacco smoking, alcohol drinking, regular exercise, BMI (continuous), use of artificial denture, hypertension, diabetes, heart disease, cerebrovascular disease, respiratory disease, digestive system diseases, cancer, eye disease, arthritis and ADL disabled. Plant-based DDS and animal-based DDS were mutually adjusted.

Supplemental Table S5. The incidence rate of cognitive impairment among DDS change patterns in subgroups

**Subgroups**	DDS change patterns from baseline to first follow up								
	High-High	High-Medium	High-Low	Medium-High	Medium-Medium	Medium-Low	Low-High	Low-Medium	Low-Low
**Age (years)**									
65~79	18.0	26.1	28.5	18.1	24.9	27.8	12.3	28.1	33.3
80~89	64.4	76.9	84.1	75.3	83.0	94.2	75.7	94.3	117.2
**Sex**									
Male	29.1	44.6	51.2	36.9	49.2	67.7	51.5	64.1	102.7
Female	42.7	53.9	59.9	53.7	64.6	68.0	47.4	73.8	84.5
**Marital status**									
Married	23.2	31.1	32.3	27.0	36.8	39.6	27.1	47.6	66.9
Not married	53.5	69.8	75.6	64.3	72.4	82.7	69.0	82.2	100.2
**Tobacco smoking**									
Current or former smoker	31.7	41.2	56.7	34.1	50.1	63.7	47.9	65.0	85.3
Non-smoker	36.7	54.2	57.2	52.1	61.5	69.6	49.6	72.5	89.8
**Alcohol drinking**									
Current or former drinker	33.9	46.1	64.5	39.3	55.5	63.8	41.4	66.8	127.8
Non-drinker	34.8	50.6	54.4	47.6	58.4	69.6	52.5	71.4	78.6
**Regular exercises**									
Yes	32.6	44.6	68.0	41.1	54.3	71.5	46.3	66.6	86.5

No	36.2	52.5	50.8	47.1	59.3	66.6	49.8	71.1	90.5
**Use of artificial denture**									
Yes	37.5	44.9	62.4	44.2	54.0	48.9	38.1	54.6	48.5
No	32.5	51.0	55.3	45.0	58.9	74.0	53.8	74.1	99.6
**ADL disabled**									
Yes	87.5	89.5	151.0	97.4	107.6	128.8	66.3	136.1	164.1
No	31.8	46.2	49.8	40.8	53.4	61.8	48.0	64.3	83.2

Incidence rate (1,000 person years).

Supplemental Table S6. Sensitivity analyses of the association between DDS change patterns and cognitive impairment

Subgroups	DDS change patterns								
	High-High	High-Medium	High-Low	Medium-High	Medium-Medium	Medium-Low	Low-High	Low-Medium	Low-Low
**Overall DDS**									
HR (95%CI) [a]	1.00 (ref)	1.31 (1.06-1.62)	1.52 (1.02-2.27)	1.09 (0.89-1.34)	1.66 (1.40-1.99)	1.90 (1.51-2.37)	1.18 (0.79-1.77)	2.51 (1.98-3.17)	2.84 (2.16-3.74)
HR (95%CI) [b]	1.00 (ref)	1.25 (1.05-1.50)	1.36 (0.95-1.95)	1.07 (0.90-1.27)	1.47 (1.26-1.71)	1.77 (1.46-2.16)	1.04 (0.72-1.50)	2.11 (1.72-2.58)	2.57 (2.00-3.30)
**Plant-based DDS**									
HR (95%CI) [a]	1.00 (ref)	1.14 (0.91-1.44)	1.59 (1.12-2.26)	0.88 (0.70-1.11)	1.33 (1.09-1.62)	1.43 (1.13-1.80)	1.13 (0.76-1.66)	1.52 (1.20-1.93)	2.02 (1.56-2.60)
HR (95%CI) [b]	1.00 (ref)	1.15 (0.95-1.39)	1.52 (1.12-2.07)	0.88 (0.72-1.07)	1.22 (1.04-1.44)	1.31 (1.07-1.60)	0.97 (0.68-1.39)	1.43 (1.16-1.75)	1.91 (1.52-2.39)
**Animal-based DDS**									
HR (95%CI) [a]	1.00 (ref)	1.27 (1.03-1.55)	1.35 (1.00-1.83)	1.17 (0.97-1.41)	1.66 (1.40-1.97)	1.80 (1.48-2.19)	1.07 (0.80-1.43)	1.77 (1.45-2.16)	1.99 (1.61-2.45)
HR (95%CI) [b]	1.00 (ref)	1.15 (0.96-1.37)	1.22 (0.94-1.58)	1.03 (0.87-1.20)	1.39 (1.20-1.61)	1.53 (1.29-1.81)	0.90 (0.71-1.15)	1.53 (1.29-1.81)	1.72 (1.43-2.07)

DDS: dietary diversity score.

[a] We excluded those with self-reported prevalent chronic diseases, including hypertension, diabetes, heart diseases, cerebrovascular diseases, respiratory diseases, digestive system diseases and cancer at baseline.

[b] We excluded those who developed cognitive impairment within the fourth year of follow-up.

Adjusted for age (continuous), sex, residence, educational level, occupation, source of income, current marital status, living pattern, tobacco smoking, alcohol drinking, regular exercise, BMI (continuous), use of artificial denture, hypertension, diabetes, heart disease, cerebrovascular disease, respiratory disease, digestive system diseases, cancer, eye disease, arthritis and ADL disabled.
